# Case Report: A Primary Right Ventricular Vascular Malformation Presenting as a Mass

**DOI:** 10.3389/fcvm.2021.736199

**Published:** 2021-10-01

**Authors:** Hongduan Liu, Xin Li, Cuiwei Zhang, Chengming Fan, Liming Liu, Juyi Wan

**Affiliations:** ^1^Department of Cardiovascular Surgery, The Second Xiangya Hospital, Central South University, Changsha, China; ^2^Department of Cardiovascular Surgery, The Affiliated Hospital of Southwest Medical University, Luzhou, China; ^3^Department of Pathology, The Affiliated Hospital of Southwest Medical University, Luzhou, China

**Keywords:** cardiac surgery, cardiac tumor, echocardiography, vascular malformation, hemangioma

## Abstract

Primary right ventricular vascular malformation is a rare primary benign anomaly in heart in nature. Due to the extremely low incidence and the progress on the classification of vascular malformation, a few cases were reported in the literatures. In the current case study, a 55-year-old women presented with a cardiac mass that was identified in right ventricle during a routine medical checkup. Magnetic resonance imaging demonstrated a well-circumscribed mass attached to the interventricular septum. Median sternotomy for the surgical resection of the mass and a cardiopulmonary bypass were performed. The intraoperative transesophageal echocardiogram showed that the mass had been successfully removed. The patient recovered well and was discharged from hospital 9 days after the surgery. The pathological diagnosis was primary cardiac arteriovenous malformation. No mass recurrence was shown by echocardiography during the 13 months' follow-up.

## Introduction

The primary cardiac arteriovenous malformation (AVM) is a rare benign vascular malformation in nature, which develops from vascular endothelial hyperplasia (1). In terms of the proportion of cardiac AVM is an extreme rarity in heart and its clinical information is limited. Herein, a case of primary cardiac AVM presenting as right ventricular mass was reported and the multimodality imaging, classification and the differential diagnosis of AVM were presented.

## Case Presetation

A 55-year-old female was referred to our hospital because a cardiac tumor in right ventricle was detected by transthoracic echocardiography during a medical checkup. The patient was asymptomatic, with a history of diabetes for 5 years and hypertension for 1 year. No cough, dyspnea, dizziness and fever were presented when she was admitted. The blood pressure was 112/56 mmHg with Irbesartan intake and the heart rate was 84 beats per min. No cardiac murmur was heard and no edema was detected in the lower limb. There were no family history of cardiovascular disease following medical history and physical examination. Laboratory tests were negative.

**Figure 1:**
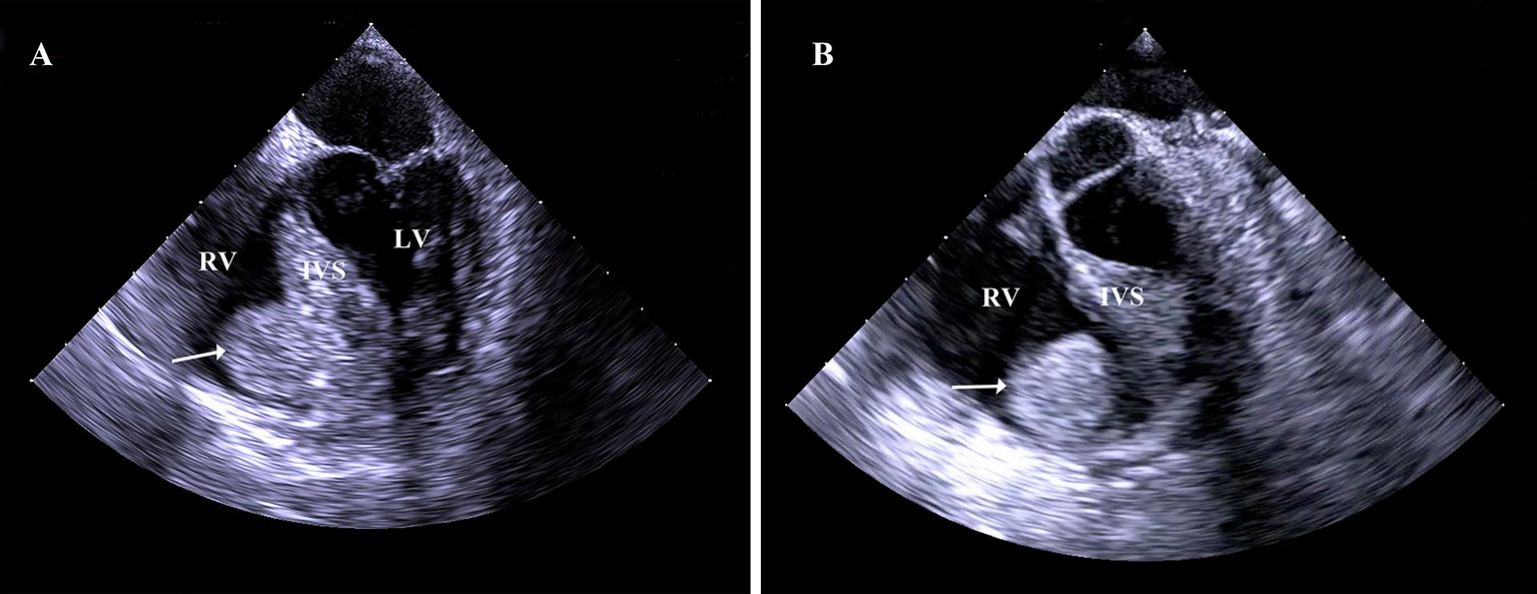
**(A,B)** Transesophageal echocardiography showed the cardiac mass (15 x 14 mm) located in right ventricle, attached to the interventricular septum abutting the apex. RV, right ventricle; IVS, interventricular septum; LV, left ventricle.

**Figure 2:**
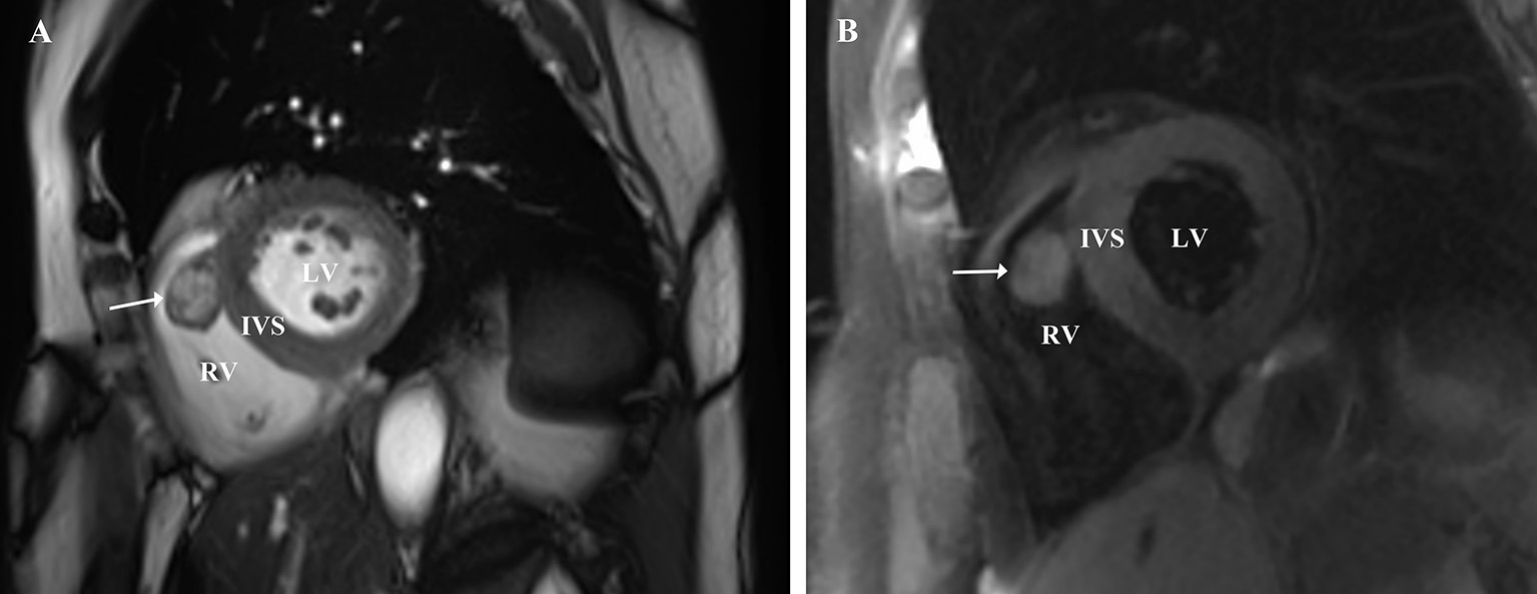
**(A,B)** Magnetic resonance imaging demonstrated that a well-circumscribed homogenous nodule, measuring 16 × 13 mm, with medium signal intensity on T1-weighted images and medium signal intensity on T2- weighted images in the right ventricle, located adjacent to the IVS, was revealed uniform moderate enhancement for the first perfusion scan and uniform significantly delayed enhancement subsequently. RV, right ventricle; IVS, interventricular septum; LV, left ventricle.

Electrocardiography was normal. Transesophageal echocardiography showed the cardiac mass (15 x 14 mm) located in right ventricle, attached to the interventricular septum (IVS) abutting the apex (Figure 1; Supplementary Video 1). A well-circumscribed homogenous “shadow,” representing a nodule measuring 16 × 13 mm, with equal T1- and T2-weighted signal intensity in the right ventricle, located adjacent to the IVS, was revealed using moderate enhancement for the first perfusion scan and delayed myocardial enhancement MRI subsequently (Figure 2). Coronary angiography (CA) showed stenosis (30%) in the right coronary artery and no obvious stenosis in the left coronary; a discernible tumor-feeding artery was not detected.

**Figure 3:**
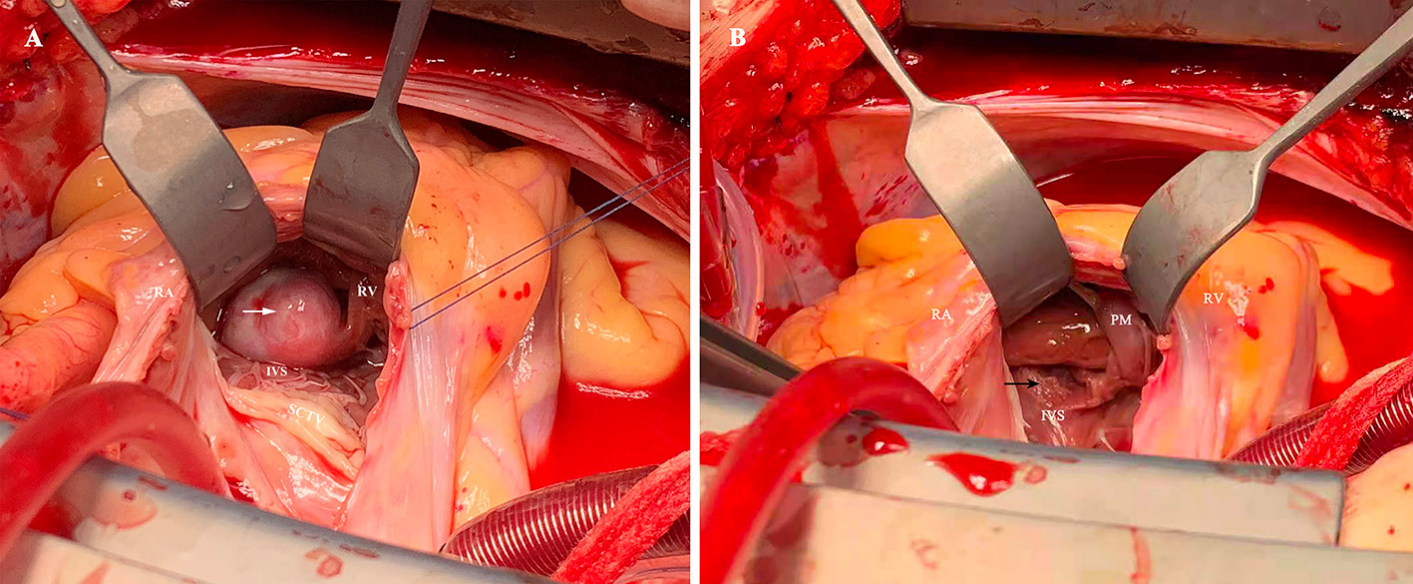
**(A)** The mass located in the right ventricle and was attached to the interventricular septum (white arrow). **(B)** The mass were removed completely and the IVS was intact without perforation (black arrow). RA, right atrium; RV, right ventricle; IVS, interventricular septum; SCTV, septal cusp of tricuspid valve; PM, papillary muscles.

**Figure 4:**
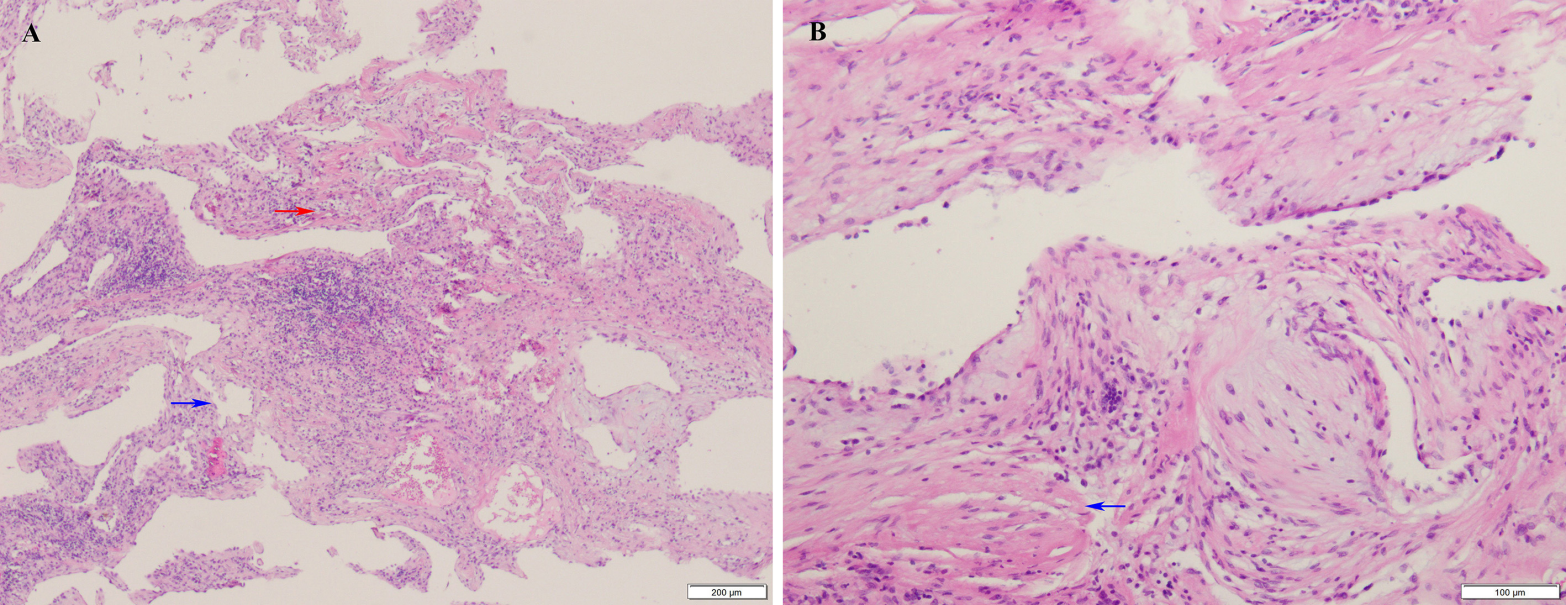
**(A)** Histopathology revealed that there are a lot of branches of vessels, thick-walled arteries (red arrow) and thin-walled veins (blue arrow), which was identified as cardiac arteriovenous malformation. **(B)** Histopathology showed the anomalous arteries with thick wall are composed of smooth muscle (blue arrow).

Initially, the clinical diagnosis was right ventricular myxoma or fibroelastoma. Median sternotomy for the surgical resection of the mass was performed to avoid the risk of pulmonary embolization and determine the tumor characteristics. A cardiopulmonary bypass was carried out using ascending aortic and superior and inferior vena cava cannulation. Upon cardiac arrest, the right atrium was opened, and it was confirmed that the semispherical mass, with soft texture and broad base, originated from the IVS (Figure 3A). Because no significant boundary was detected preoperatively, the mass along with a small portion of IVS was resected (Figure 3B). The resected specimen comprised a reddish mass enveloped with blood. Signs of ventricular septal defect were not found with TTE (Supplementary Figure 1). The histopathological examination revealed that the tumor was a cardiac AVM (Figures 4A,B). The patient's postoperative course was uneventful. She was shown to have recovered well and without recurrence at the 13-month follow-up.

## Disscusion and Conclusion

The AVM was considered as a benign vascular tumor, some of which was termed as plexiform hemangioma in the past (2). However, the classification was changed since the International Society for the Study of Vascular Anomalies (ISSVA) grouped the vascular anomalies into two major categories, vascular tumors (mainly hemangiomas) and vascular malformations; According to this classification, AVM is a vascular malformation subtype (3, 4). In our case, the right ventricular mass was identified as the AVM which is extreme rare in the limited literature.

The differential diagnosis of a mass attached to the right ventricle includes myxoma, lipoma, fibroelastoma, and hemangioma. Categorization of the symptoms depends on their location and the pathological classification of the mass. Some cases are characterized by pericardial effusion, right ventricular outlet obstruction, and even sudden cardiac death (5–7). The location of cardiac benign masses varies, typically involving the atrium, ventricle, aortic valve, mitral valve, or epicardium (8, 9). In the present case, the patient was asymptomatic, and the mass was attached to the IVS in the right ventricle, abutting the apex. Initially, fibroelastoma and cardiac myxoma were suspected.

Appropriate screening imaging modalities for cardiac mass include echocardiography, CT, and MRI, all of which can be used to eliminate the problem of a differential diagnosis (10, 11). In the current study, a diagnosis of benign tumor was made, based on the MRI and echocardiographic findings. Enhanced myocardial echocardiography and ^18^F-fluorodeoxyglucose-positron emission tomography can also be utilized to provide detailed information to resolve a differential diagnosis (12, 13). In addition, CA can be used to determine whether a coronary artery is feeding the tumor; however, it should only be considered if performing CA is consistent with the patient's age and symptoms (14). Although echocardiography, CT or MRI can be helpful in the diagnosis and differential diagnosis, the histopathology is still needed for the final diagnosis. In the current case study, a right ventricular mass was identified using TE and intraoperative TEE, which showed its location, diameter, and morphology; it also showed that the IVS was intact following the resection. TEE and MRI clearly showed the mass' margin and feeding artery to the mass, which informed the surgeons of the confidence to completely remove it. In the event that the patient presents with symptoms caused by the mass or in cases when the prognosis is uncertain in relation to the tumor, surgical resection should be performed to reduce the risk of embolism (15). Conservative management is an option when the biopsy returns a pathological classification of vascular malformation, except in the case of concomitant aortic valve insufficiency (9).

The ISSVA classification standards have been published; however, there is a lack of consensus regarding the understanding of whether vascular anomalies are tumors or malformations (16). Initially, they were classified according to factors such as vascular distortion and structure. Recently, an analysis of WT-1 and GLUT-1 expression is used to distinguish between vascular malformation and vascular tumors (17, 18). Elsewhere, a MSOT-based, non-invasive assessment of hemoglobin levels was used to differentiate between vascular malformation subtypes (19). In addition, advances in molecular genetics are ensuring greater insight into the genetic basis for vascular anomalies and providing potential molecular targets for pharmacotherapy. Thus, in future, vascular malformations may be treated using novel pharmacotherapeutic approaches rather than surgery (3).

Right ventricular AVM is extremely rare which might be resulted in sudden death. Currently, echocardiography is the primary and most common method for detecting ventricular AVM as it effectively indicates its location, diameter, and morphology. Likewise, multiple imaging plays a key role in solving the problem of a differential diagnosis, while providing invaluable information to assist with complete resection of the mass. Although echocardiography, CT or MRI can be helpful in the diagnosis, it is still challenging. After all, the histopathology is still the “gold standard” in the final diagnosis.

## Data Availability Statement

The original contributions presented in the study are included in the article/Supplementary Material, further inquiries can be directed to the corresponding author/s.

## Ethics Statement

The study protocol was approved by the Ethics Committee of the Affiliated Hospital of South West University, Luzhou, China. The patients/participants provided informed consent to participate in this study. Written informed consent was obtained from the individual(s) for the publication of any potentially identifiable images or data included in this article.

## Author Contributions

HL drafted the manuscript. HL and JW designed the study. XL, HL, and JW performed the surgery and were responsible for the collection of data or analysis. JW, CF, and LL revised the manuscript. CZ provided the pathological outcome. All have authors read and approved the final manuscript.

## Funding

This study was supported in part by the following funding sources: Sichuan Province science and technology projects (2018JY0405, 2021YFH0148, and 2020YJ0190), Science and Technology Strategic Cooperation Programs of Luzhou Municipal People's Government and Southwest Medical University (2019LZXNYDJ30).

## Conflict of Interest

The authors declare that the research was conducted in the absence of any commercial or financial relationships that could be construed as a potential conflict of interest.

## Publisher's Note

All claims expressed in this article are solely those of the authors and do not necessarily represent those of their affiliated organizations, or those of the publisher, the editors and the reviewers. Any product that may be evaluated in this article, or claim that may be made by its manufacturer, is not guaranteed or endorsed by the publisher.

## Supplementary Material

Supplementary Video 1 Transesophageal echocardiography showed the cardiac mass located in right ventricle, attached to the interventricular septum abutting the apex.

Supplementary Figure 1 Postoperative transthoracic echocardiography showed that there was no shunt across the IVS. IVS, interventricular septum.

## References

[1] McCuaig CC. Update on classification and diagnosis of vascular malformations. Curr Opin Pediatr. (2017) 29:448–54. 10.1097/MOP.0000000000000518 28654575

[2] Fan J Liao X Zhou X A. case report of primary cardiac capillary hemangioma. Cancer Biol Ther. (2016) 17:11–3. 10.1080/15384047.2015.1109391 26650115 PMC4847828

[3] Martinez-Lopez A Salvador-Rodriguez L Montero-Vilchez T Molina-Leyva A Tercedor-Sanchez J Arias-Santiago S. Vascular malformations syndromes: an update. Curr Opin Pediatr. (2019) 31:747–53. 10.1097/MOP.0000000000000812 31693582

[4] ISSVA Classification of Vascular Anomalies ©2018 International Society for the Study of Vascular Anomalies. (2018). Available online at: https://www.issva.org/UserFiles/file/ISSVA-Classification-2018.pdf (accessed September 23, 2016).

[5] Rathore K Yussouf R Teh M Jindal S Wong D Newman M. Left atrial anastomosing hemangioma causing recurrent pericardial effusion. Ann Thorac Surg. (2020) 109:e157–9. 10.1016/j.athoracsur.2019.06.082 31430463

[6] Wildgruber M Sadick M Muller-Wille R Wohlgemuth WA. Vascular tumors in infants and adolescents. Insights Imaging. (2019) 10:30. 10.1186/s13244-019-0718-6 30868300 PMC6419671

[7] Aguilera B Suárez-Mier M Argente T. Cardiac arteriovenous malformation causing sudden death. Cardiovasc Pathol. (2004) 13:296–8. 10.1016/j.carpath.2004.06.002 15358345

[8] Kotoulas C Georgiou C Grapatsas K Kotoulas S Theodosiadis N Panagiotou I. Cavernous hemangioma of the left atrium: a rare tumor. J Card Surg. (2020) 35:202–3. 10.1111/jocs.14373 31765014

[9] Cotier P Bruneval P Amemiya K. Vascular malformation in a bicuspid aortic valve. Cardiovasc Pathol. (2019) 38:39–41. 10.1016/j.carpath.2018.10.006 30447516

[10] Gao Y Wu W Zhang L Sun Z Xie Y Li Y. Multimodality imaging in preparation for resection of a right atrial cavernous hemangioma. Echocardiography. (2020) 37:465–6. 10.1111/echo.14615 32077510

[11] Vodovar N Seronde MF Laribi S Gayat E Lassus J Boukef R. Post-translational modifications enhance NT-proBNP and BNP production in acute decompensated heart failure. Eur Heart J. (2014) 35:3434–1. 10.1093/eurheartj/ehu314 25157115

[12] Xiachuan Q Xuebin L Yongjie W. Case of cardiac hemangioma diagnosed by myocardial contrast echocardiography. Circ Cardiovasc Imaging. (2019) 12:e008811. 10.1161/CIRCIMAGING.118.008811 31030538

[13] Matsuba T Hisashi Y Yotsumoto G Imoto Y A. rare cardiac haemangioma in the right ventricle diagnosed accurately using (1)(8)F-fluorodeoxyglucose-positron emission tomography. Eur J Cardiothorac Surg. (2015) 47:e223–5. 10.1093/ejcts/ezu540 25602049

[14] Chen X Lodge AJ Dibernardo LR Milano CA. Surgical treatment of a cavernous haemangioma of the heart. Eur J Cardiothorac Surg. (2012) 41:1182–3. 10.1093/ejcts/ezr153 22219444

[15] Rekik S Hentati M Boudawara T Abdennadher M Frikha I Kammoun S. Myofibroblastic tumor of the right ventricle causing bilateral pulmonary embolism in a 31 year-old woman. Int J Cardiol. (2009) 131:e131–3. 10.1016/j.ijcard.2007.07.082 17967491

[16] Pahl KS Kim K Sams C Alvarez H Smith SV Blatt J. Inconsistency in classifying vascular anomalies: what's in a name? Pediatr Blood Cancer. (2018) 65. 10.1002/pbc.26836 28988459 PMC6015739

[17] Al Dhaybi R Powell J McCuaig C Kokta V. Differentiation of vascular tumors from vascular malformations by expression of Wilms tumor 1 gene: evaluation of 126 cases. J Am Acad Dermatol. (2010) 63:1052–7. 10.1016/j.jaad.2009.12.017 21093662

[18] Rastogi K Singh L Khan NA Goyal S Khatri A Gupta N. Benign vascular anomalies: a transition from morphological to etiological classification. Ann Diagn Pathol. (2020) 46:151506. 10.1016/j.anndiagpath.2020.151506 32200223

[19] Masthoff M Helfen A Claussen J Karlas A Markwardt NA Ntziachristos V. Use of multispectral optoacoustic tomography to diagnose vascular malformations. JAMA Dermatol. (2018) 154:1457–62. 10.1001/jamadermatol.2018.3269 30267083 PMC6583374

