# Supplementary figures and images for: Case Report: A Primary Right Ventricular Vascular Malformation Presenting as a Mass
Source: Front Cardiovasc Med. 2021 Oct 1;8:736199. doi: 10.3389/fcvm.2021.736199 (PMC8517140; doi:10.3389/fcvm.2021.736199)

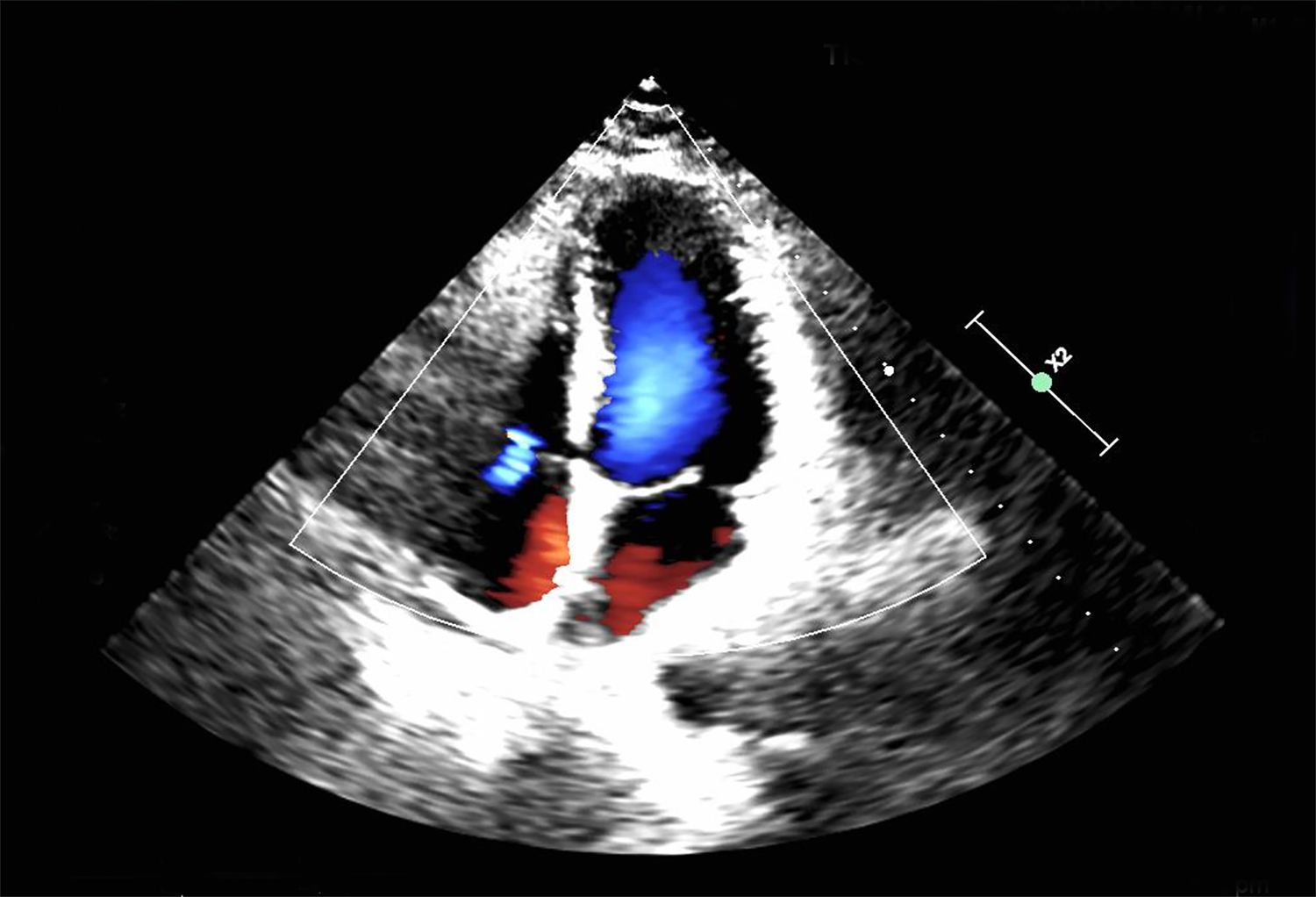

Supplement: Supplementary Figure 1 — Postoperative transthoracic echocardiography showed that there was no shunt across the IVS. IVS, interventricular septum. [file Image_1.jpg]
